# Determinant of Implanon Discontinuation among Women Who Ever Used Implanon in Diguna Fango District, Wolayita Zone, Southern Ethiopia: A Community Based Case Control Study

**DOI:** 10.1155/2017/2861207

**Published:** 2017-11-06

**Authors:** Amanuel Tadesse, Mekides Kondale, Eskzyiaw Agedew, Feleke Gebremeskel, Negussie Boti, Bilcha Oumer

**Affiliations:** ^1^Department of Public Health, College of Medicine and Health Sciences, Arba Minch University, Arba Minch, Ethiopia; ^2^Department of Midwifery, College of Medicine and Health Sciences, Arba Minch University, Arba Minch, Ethiopia; ^3^Department of Nursing, College of Medicine and Health Sciences, Arba Minch University, Arba Minch, Ethiopia

## Abstract

**Background:**

A significant number of women make Implanon their first choice of contraception. However, they discontinue their Implanon before its expiry date was high, but factors that contribute to discontinuing their Implanon were poorly described in Ethiopia.

**Methods:**

A community based unmatched case control study was conducted. Then simple random sampling technique was used to select 340 women. Data was collected by nurses using face to face interview. Epi-Info version 7 and SPSS 20 software were used. Bivariate and multiple logistic regressions were performed with COR and AOR with 95% CI.

**Findings:**

Having preinsertion counseling (AOR: 0.36, 95% CI: 0.20–0.64), having follow-up appointment (AOR: 0.35, 95% CI: 0.2–0.62), age at insertion <20 years (AOR: 3, 95% CI: 1.16–7.8), women who had no formal education (AOR: 2.8, 95% CI: 1.31–6.11), women who had ≤4 children (AOR: 1.8, 95% CI: 1.01–3.21), and women who had previous abortion history (AOR: 2.3, 95% CI: 1.10–4.63) were determinants of Implanon discontinuation.

**Conclusions:**

Policy makers and concerned bodies should take into account future intervention and also great emphasis should be given to follow-up appointment and counseling services, especially counseling on side effects, and informed choice for clients after Implanon insertion.

## 1. Background

Implanon is Long-Acting Reversible Contraceptive and extremely effective at preventing pregnancy with a clinical failure rate of less than 1% [1–3]. Its main mechanism of action is ovulation suppression, augmented by increased cervical mucus viscosity that hinders the passage of spermatozoa and alters the endometrial lining [2, 4].

Although it involves minor surgical procedures, the woman should be adequately counseled, including the usual information on general advantages and disadvantages of implants. The counseling should also include offering the woman the right to discontinue Implanon use at any time, information that implant site-related adverse events could occur, as well as clarification of the rapid return to fertility once the implant is removed [5]. The first implant, Norplant, was licensed in 1983 although global production was discontinued in 2008 after a lawsuit accused Norplant of causing scarring, pain upon removal, and other side effects. Newer generations of implants are smaller and easily inserted and removed and have fewer complications. These include Implanon, Jadelle, Norplant, and Sino Implant [1].

More than 4.5 million women have used Implanon worldwide [4, 6]. In sub-Saharan Africa, a growing number of women and sexually active adolescents are using family planning, and many are choosing contraceptive implants [7]. As the Ethiopian Demographic and Health Survey (EDHS) 2016 reported the implant users are only about 8% among all method users [8]. Southern Nations Nationalities and Peoples Regional State (SNNPRS) was one of the regions where implant utilization was 8% [8].

Evidence from studies conducted in Egypt, Kenya, Malawi, Zimbabwe, and Ethiopia revealed that women implant is their first choice, but percentage of early discontinuation of Implanon ranges from 17% to 47% [9, 10]. Ethiopia has made considerable strides in providing access to FP services. In order to ensure the delivery of primary health services throughout the country, the Federal Ministry of Health (FMoH) of Ethiopia Government committed to enhance the Reproductive Health (RH) status of women, men, and young people of Ethiopia through the four-tiered health care delivery system [9, 11–13]. In addition to this the FMoH had also been working with different partners since 1995, to improve RH/FP services both at community and at facility levels [14, 15].

In 2003, Ethiopian MoH launched the health extension program (HEP), which was intended to increase access to RH care [16, 17]. Despite the fact that the Ethiopian government had been working over the past decades to improve RH services and making health facility services accessible and usable for all reproductive age women, encouraging contraceptive continuation has been of less priority than encouraging new adopters in the country [18]. Implanon discontinuation remains unacceptably high in different parts of Ethiopia [11, 19, 20]. High rates of contraceptive discontinuation for reasons other than the reduced needs for contraception are public health concern because of their association with negative RH outcomes [10, 21].

Evidence from different countries indicated that the discontinuation of contraceptive use places a woman at risk of unintended pregnancy, which in turn leads to potentially unsafe induced abortions where access to safe abortion is restricted, births that pose a risk to the health of the mother and child, and ultimately reduced educational attainment of children that may lead to the fact that family planning programs can have only limited impact on fertility reduction as well as being unintentionally pregnant [1, 10, 19].

Therefore currently, little is known about the determinants of discontinuation of Implanon among women who use Implanon in Ethiopia as well as in the study area. The aim of this study was to identify the determinants of Implanon discontinuation among women in Diguna Fango District, Southern Ethiopia.

## 2. Methods and Materials 

### 2.1. Study Area and Study Design

This study was conducted in Diguna Fango District from March 20 to April 20, 2017. Diguna Fango District is one of the 12 rural districts in Wolayita Zone. The District's capital town Bitena is located at 430 km from Addis Ababa and 47 kilometers far from Sodo, capital of Wolayita Zone. It is administratively divided into 26 rural and 6 semiurban kebeles. According to central statistics agency (CSA) 2007, there were 121,040 populations and 24,702 households with women of reproductive age group (15–49) and 27,839 in 2015. There are 5 government health centers with one primary hospital and 31 health posts which provide FP services. In all the five health centers, the primary hospital has relatively similar composition of health professionals with the same level of health service provision. Moreover, the 31 health posts have at least two health extension workers who are providing the same standard of health care services including Implanon insertion. According the District report Implanon coverage was 28.9% (report from Diguna Fango District Health Office, December 2016). A community based* case control* study design was used to identify the determinants of Implanon discontinuation among women who ever used Implanon.

### 2.2. Source and Study Populations

All women of reproductive age group (15–49) who ever used Implanon in Diguna Fango District were source population: cases were all women of reproductive age in the selected kebele who have discontinued their Implanon before 3 years of insertion and controls were all women of reproductive age group who used Implanon for the complete 3 years in selected kebeles that meet the inclusion criteria.

### 2.3. Sample Size Determination and Sampling Procedures

The following assumptions were made in calculating sample size: 95% confidence level, 80% power, and minimum detectable OR of 2.79. Side effect was used as the exposure variable. The proportion of case with side effect was 87.4% as well as 71.4% of cases [11]. Sample size was calculated using STATCALC program of Epi-Info statistical package version 7 with a case to control the ratio of 1 : 3. After adding 10% nonresponse, total sample size was 340, including 85 for cases and 255 for controls. Simple random sampling was used to select study participants using the master family index as the sampling frame. Diguna Fango District has a total of 32 kebeles, the lowest administrative unit in Ethiopia, and 25% kebeles were included in the study (eight kebeles). Sample was allocated proportionally to population across 8 kebeles based on the number of women who ever used Implanon in the six months preceding the survey. The study subjects were selected by using simple random sampling techniques.

### 2.4. Operational Definition

*Implanon*. A one-rod implant contraceptive was inserted under the skin of a woman's upper arm that is effective for three years in preventing pregnancies and contains the hormone etonogestrel.

*Do Not Discontinue Implanon*. It is not discontinuation of use of Implanon before 3 years after insertion of Implanon.

*Implanon Discontinuation*. It is discontinuation of use of Implanon before 3 years after insertion of Implanon.

### 2.5. Data Collection Procedure and Data Quality Control

A semistructured, pretested questionnaire was adapted from EDHS 2011 and other published literatures. The questionnaire was prepared in English, translated to the local language (Wolayttatto doona), and back translated to English by language experts to ensure consistency. The questionnaire which has four parts was sociodemographic. Discontinuation while still in need, counseling service, and obstetric related factors were used. Pretest was done two weeks before the survey in nearby District kebele, on 17 (5%) of the sample (5 cases and 12 controls). Based on the pretest, a questionnaire was corrected to ensure clarity, wording, and logic sequence and skip patterns. Data was collected by eight diploma trained health professionals and supervised by two B.S. trained health professionals. All data collectors and supervisors were trained adequately for one day and performed practical exercises to be familiar with the questionnaire.

### 2.6. Data Processing and Analysis

The collected data were coded, cleaned, and entered by Epi-Info version 3.5.1 and exported to statistical package for social science (SPSS) version 20.0 for analysis. Descriptive statistics including graphs, charts, tables, and proportion was used to describe the data. Bivariate and multivariable logistic regression analysis was performed to see the association between outcome and explanatory variables. Variables that were found to be statistically significant in the bivariate analysis (*p* < 0.25) were enter into multivariable logistic regression model. Finally multivariable logistic regression analysis was done to identify factors associated with Implanon discontinuation. A *p* value ≤ 0.05 was considered statistically significant in this study. An effort was made to assess whether the necessary assumptions for the application of multivariable logistic regression were fulfilled. In this regard, the Hosmer and Lemeshow's goodness-of-fit test with large *p* value (*p* > 0.05) was checked to see good fitness. This statistic was computed as the Pearson chi-square from the contingency table of observed frequencies and expected frequencies. Multicollinearity and confounding effect was checked by using standard error. The variable without multicollinearity was entered into multivariable model. Only variable with *p* < 0.05 were reserved in the final model. Odds ratio along with 95% confidence interval (CI) was used to assess the association between explanatory variables and Implanon discontinuation. Level of statistical significance was declared at *p* value less than 0.05.

### 2.7. Ethical Considerations

The study protocol was approved by the Ethical Review Committee (ERC) of College of Medicine and Health Science, Arba Minch University. Based on the approval, an official letter was written by AMU Public Health Department to Zonal Health Department. Explanation on the objective of the research was provided to the concerned personnel at Zonal level. Support letter that was submitted to the District Health Office was obtained from the Zonal Health Department. Similarly, the District wrote the letter to the health facilities and kebeles for cooperation. At last data were collected after assuring the confidentiality nature of responses and obtaining oral consent from the study participant. All the study participants were encouraged to participate in the study and at the same time they have also been told that they have the right not to participate.

## 3. Results

### 3.1. Sociodemographic Characteristics of the Study Participants

A total of 340 respondents (85 cases and 255 controls) participated in the study. The mean (±SD) age of the study participants was 32.5 (±4.9) years: 26.6 (±4.4) for cases and 34.5 (±3.2) years for controls with age ranging from 19 to 42 years. About 49 (57.6%) of cases and 177 (69.4%) controls were from rural areas. Majority of cases, 65 (76.5%), and controls, 228 (89.4%), were protestant. Majority of cases had no formal education (46, 54.1%) cases as well as 84 (32.9%) controls. Thirty-nine (45.9%) cases and 117 (45.9%) controls were housewives by occupation. Approximately half (45.9%) of cases' husbands were illiterate. Eighty-seven (34.1%) controls husbands had attended grades one to eight. Around 28.2% of cases and 60.4% of controls did not have a radio or television (Table 1).

### 3.2. Family Planning Service Related Characteristic of the Study Participants

Regarding contraceptive information, 70 (82.4%) of cases and 164 (64.3%) of controls heard about contraceptives from health extension workers. Majority of cases 49 (57.6%) and controls 139 (54.5%) never used contraceptives before Implanon. The dominant contraceptives ever used before Implanon were injectable (25 (29.4%) among cases and pills and 128 (50.2%) among controls). Concerning counseling service, only about 31 (36.5%) of cases and 167 (65.5%) of controls obtained counseling service before Implanon insertion. This study also revealed that 198 (58.2%) of the study participants, 31 (36.5%) of cases, and 167 (65.5%) of controls obtained counseling service before Implanon insertion. Similarly, about 40 (47.1%) of cases and 160 (62.7%) of controls reached a decision themselves on Implanon use. Among those provided counseling services, the majority of cases 25 (29.4%) and 74 (29%) of controls get individual counseling. Moreover, 40 (72.7%) of cases and 65 (56%) of controls were counseled on average. In about 45 (52.9%) cases and 95 (37.3%) controls, decision to use Implanon was made by providers and their husbands. A large number of cases 58 (68.7%) and controls 173 (67.8%) had Implanon inserted in health centers and hospital (Table 2).

### 3.3. Obstetric Related Characteristic of the Study Participants

This study also found the large proportion of cases 47 (55.5%) and (57.3%) controls 146 were having more than 4 children and ≤4 children, respectively. Of 85 cases that discontinued their Implanon before 3 years, 36 (42.4%) women responded that they experienced pregnancy after Implanon discontinuation. Among 36 women who experienced pregnancy after Implanon discontinuation, 21 (24.7%) responded that their pregnancy was intentional and 15 (17.6%) were told that their pregnancy was mistimed (unintentional).

### 3.4. Discontinuation While Still in Need Related Characteristic of the Study Participants

**Figure 1:**
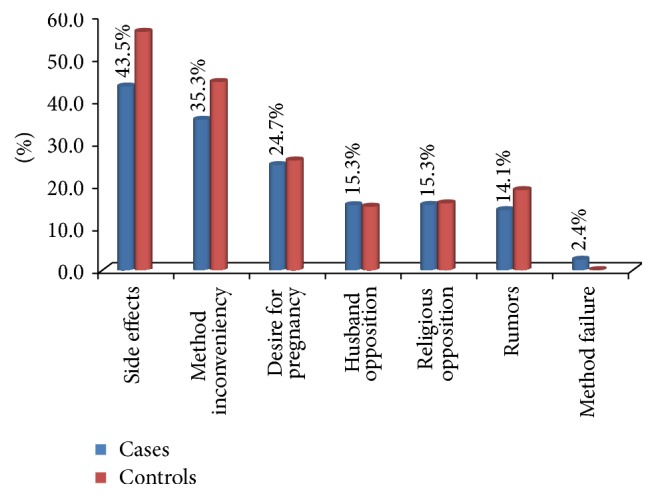
Reason of discontinuation in women who ever used Implanon in Diguna Fango District, Wolayita Zone, Southern Ethiopia, March 2017.

Among the study participant discontinuation while still in need, the majority discontinue due to side effect 37 (43.5%), method inconveniency 30 (35.5%), desire to have more children 21 (24.7%), husband opposition 13 (15.3%), rumor 12 (14.1%), and religious opposition 13 (13.5%). Moreover, among cases and controls majority of cases 37 (43.5%) and controls 143 (56.1%) experienced side effect. The major side effect was menstrual disruption 26 (70.2%) and others were headache 24 (64.8%), amenorrhea 10 (27%), weight gain 9 (24%), and other minor side effects 5 (13.5%) (Figure 1).

### 3.5. Factors Associated with Implanon Discontinuation among Women

After adjusting for other variables, women who had no formal education had increased odds of Implanon discontinuation compared to women who attended secondary school and above (AOR: 2.8, 95% CI: 1.31–6.11). Similarly, women of age category of less than 20 years had increased odds of Implanon discontinuation compared to women of age category greater than 35 years (AOR: 3, 95% CI: 1.16–7.80). The odds of Implanon discontinuation were 2.3 times greater among women who had history of previous abortion as compared to women who did not experience abortion (AOR: 2.3, 95% CI: 1.11–3.21). The odds of Implanon discontinuation were 1.8 times greater among women who had living children less than 4 as compared to women who had 4 or more living children (AOR: 1.8, 95% CI: 1.01–3.21). This study found that women who got counseling service and follow-up appointment were negatively associated with Implanon discontinuation. Those who have counseling service were 64% less likely to discontinue when compared with those without counseling service (AOR: 0.36, 95% CI: 0.21–0.64). Similarly those who have follow-up appointment given to them after Implanon insertion were 65% less likely to discontinue when compared with those without follow-up appointment (AOR: 0.35, 95% CI: 0.21–0.62) (Table 3).

## 4. Discussion

In order to effectively tackle the unreserved family planning discontinuation and its associated problems in Ethiopia in general and in study area in particular, factors influencing Implanon discontinuation need to be investigated. In this study, preinsertion counseling, follow-up appointments given after insertion, having number of living children less than four, history of abortion, and age category being less than 20 years all had higher odds of Implanon discontinuation among women who ever used Implanon.

In this study, the odds of Implanon discontinuation were three times higher for women with age category of less than 20 years compared to those with age category of >35 years. This finding is consistent with previous study conducted in Arsi Zone, Oromia, which shows that younger women with age category less than twenty were more likely to discontinue their Implanon than the older ones with age category more than thirty [22]. However, this finding is inconsistent with the study done in Debre Markos Town, Northwest Ethiopia, which revealed that age failed to be statistically significant in multivariable analysis [23]. This variation may be explained in three ways; first it might be associated with this study majority being young which means less than 24 years and those with high probability with desire to have more children which in turn leads to high discontinuation of Implanon. The second reason might be due to inadequate preinsertion counseling particularly about the expected side effects of the method; it could also result in earlier removal of Implanon while still in need. The third reason might be the difference in denominator of both studies. In this study the denominator was the sum of those women who discontinued their Implanon before 3 years and those women who used their Implanon for 3 complete years. But the study done in Debre Markos Town used all Implanon users as denominator.

This study also found that women not having formal education were three times more at risk to discontinue their Implanon as compared to women with secondary level education and above. This finding is consistent with the study undertaken on factors associated with Implanon discontinuation among women who ever used Implanon in Ofla District of Tigrai region that showed that Implanon discontinuation was more common among women not formally educated [24]. However, this finding is different from the study found in Debre Markos Town of Amhara region that revealed women having their educational level college and above were two times more likely to discontinue their Implanon than those women who had no formal education [23]. The possible explanation of this inconsistency could be the difference in reference category we used, but they used not formally educated women as reference category. After reading different literatures we used those women with secondary level education and above as reference group because these groups were at less risk of discontinuation than those who had no formal education. The possible explanation for this finding might be that women who had their secondary level education and above had better knowledge and awareness towards modern contraception than those women having no formal education; hence good continuation of Implanon might be observed among educated women.

In this study, the odds of Implanon discontinuation was two times higher for women who had number of living children less than four when compared to those women who had more than four living children. This finding was supported by the study conduct in Agarfa District which revealed that for a single increase in family size the likelihood of modern contraceptive discontinuation decreased by 12% [25]. The possible reason for this might be those women who had less than or equal to four children with desire to have more children, who hence discontinued their Implanon. The other possible explanation might be poor preinsertion counseling especially on available contraceptives and expected side effect can also result in discontinuation.

History of abortion was significantly associated with Implanon discontinuation in the study. The odds of Implanon discontinuation were considerably higher among women had previous history of abortion when compared to their counterparts. This result was consistent with a study in Australia [26]. Women had history of abortion might be the fear of childlessness in double burden of abortion and contraception.

Preinsertion counseling to family planning use has shown a significant association with Implanon discontinuation in this study. Those women who got preinsertion counseling were 64% less likely to discontinue Implanon as compared to their counterparts. This means about 64% of Implanon discontinuation prevented appropriate preinsertion counseling. This finding is supported by other similar studies done in Northern Ethiopia, Southeast Ethiopia, and Northwest Ethiopia [22, 24, 25]. The possible explanation for this might be that if women were given preinsertion counseling especially on all available methods and existing side effects, they decide on which method was better to use and good continuation might be exist. Due to this reason women who were well informed on the possible side effect of the method will tolerate minor changes, but those who were not informed seek removal of the method.

This study indicated a significant association between follow-up appointments given after insertion with Implanon discontinuation. Women who got follow-up appointments given after insertions were 65% less likely to discontinue Implanon as compared to their counterparts. This means about 65% of Implanon discontinuation could be prevented if we give appropriate follow-up appointment after insertion. This finding was also supported by other studies done in Tigri region and Amhara regions which showed that follow-up appointment for check-up was significantly associated with Implanon discontinuation [23, 24]. The possible explanation for this might be at follow-up check-up time where they might get further counseling on side effects and support from health professionals and hence might be encouraged to continue their Implanon. The other reason might be clients with a complaint of side effect especially those who suffer from menstrual disruption who could get supportive treatment from health care providers during follow-up time; hence they could extend the use of Implanon. The limitation of this study was recall bias and social desirability bias which might have led to under- or overreporting. To minimize such biases, clarification of potential ambiguities and misunderstandings and maintaining privacy of participants were carried out by interviewers. The study did not assure causality due to the nature of the design. Another limitation of this study was that it did not ascertain the providers' attitudes and quality of FP service provision. Despite these limitations, the study believes that these findings might be a reasonable source of information for researchers and program managers.

## 5. Conclusions

Having preinsertion counseling and follow-up appointments were major determinants of Implanon discontinuation. Other determinants found to be significantly associated with Implanon discontinuation were women's age at Implanon insertion less than 20 years, women educational status, living children of ≤4, and previous history of abortion.

## Figures and Tables

**Table 1:** Sociodemographic characteristics of study participants among women who ever used Implanon in Diguna Fang District, Wolayita Zone, Southern Ethiopia, March 2017.

Variables		Cases (*n* = 85)	Controls (*n* = 255)
		*n* (%)	*n* (%)
Age of respondents in years	<20	10 (11.8)	70 (27.5)
	20–24	24 (28.2)	68 (26.7)
	25–29	16 (18.8)	37 (14.5)
	30–34	16 (18.8)	40 (15.7)
	35+	19 (22.4)	40 (15.7)

Residence of respondents	Semiurban	36 (42.4)	78 (30.6)
	Rural	49 (57.6)	177 (69.4)

Religion of respondents	Protestant	60 (70.6)	220 (86.3)
	Orthodox	13 (15.3)	20 (7.8)
	Catholic	7 (8.2)	9 (3.5)
	Muslim	5 (5.9)	6 (1.8)

Ethnicity of respondents	Wolayita	73 (85.9)	228 (89.4)
	Sidama	5 (5.9)	4 (1.6)
	Amhara	5 (5.9)	7 (2.7)
	Oromo	2 (2.4)	16 (6.3)

Maternal educational level	Having no formal education	38 (44.7)	62 (24.3)
	Elementary	26 (30.6)	78 (30.6)
	Secondary and above	21 (24.7)	115 (45.1)

Maternal occupation	Housewife	39 (45.9)	117 (45.9)
	Merchant	29 (34.1)	99 (38.8)
	Employed	17 (20.0)	39 (15.3)

Husband educational level	Having no formal education	40 (47.1)	153 (60.0)
	Elementary	32 (15.3)	59 (23.1)
	Secondary and above	13 (15.3)	43 (16.9)

Number of children	1–4 children	38 (44.7)	146 (57.3)
	≥5 children	47 (55.3)	109 (42.7)

History of abortion	Yes	15 (17.6)	73 (28.6)
	No	70 (82.4)	182 (71.4)

**Table 2:** Family planning service related characteristic of women who ever used Implanon in Diguna Fango District, Wolayita Zone, Southern Ethiopia, March 2017.

Variables	Categories	Cases (*n* = 85)	Controls (*n* = 255)
		*n* (%)	*n* (%)
Ever used contraceptives before Implanon	Yes	49 (57.6)	139 (54.5)
	No	36 (42.4)	116 (45.5)

Source of information	Friends	9 (10.6)	7 (2.7)
	Health extension worker (HP)	70 (82.4)	164 (64.3)
	Health worker (HC)	5 (5.9)	82 (32.2)
	Media/TV/radio	1 (1.2)	2 (0.8)

Ever used contraceptives before	Pills	24 (28.2)	128 (50.2)
	Injectables	25 (29.4)	106 (41.6)
	Jadelle	0 (0.0)	3 (1.2)
	IUCD	0 (0.0)	4 (1.6)

Obtained counseling service before Implanon insertion	Yes	31 (36.5)	167 (65.5)
	No	54 (63.5)	88 (34.5)

Type of counseling given	Individual counseling	25 (29.4)	74 (29)
	Mass counseling	9 (10.6)	21 (8.2)
	With husband counseling	18 (21.2)	20 (7.8)

Information obtained during counseling	Advantage	31 (100)	165 (98.8)
	Duration of action	31 (100)	167 (100)
	Side effect	14 (45)	140 (83.8)
	Effectiveness	31 (100)	165 (98.8)
	Informed choice	12 (38.9)	150 (89.9)

Decision maker to use Implanon	Woman herself	40 (47.1)	160 (62.7)
	Other^*∗*^	45 (52.9)	95 (37.3)

Place of insertion	Health post	27 (31.8)	82 (32.2)
	Health center and hospital	58 (68.7)	173 (67.8)

*Key note*.  ^*∗*^Provider, husband.

**Table 3:** Factors associated with discontinuation of Implanon among women who ever used Implanon in Diguna Fango District, Wolayita Zone, Southern Ethiopia, March 2017 (*n* = 340).

Variables	Cases number (%)	Controls number (%)	COR (95% CI)	AOR (95% CI)	*p* value
*Place of residence*					
Semiurban	36 (42.4)	78 (30.6)	1	1	
Rural	49 (57.6)	177 (69.4)	1.7 (1.01–2.77)^*∗*^	1.5 (0.84–2.71)	0.171
*Age at insertion*					
<20 years	10 (11.8)	70 (27.5)	3.3 (1.41–7.85)^*∗*^	3 (1.16–7.80)^*∗∗*^	0.023
20–24	24 (28.2)	68 (26.7)	1.35 (0.66–2.76)	1.1 (0. 45–2.31)	0.961
25–29	16 (18.8)	37 (14.5)	1.01 (0.49–2.45)	1.1 (0.46–3.07)	0.717
30–34	16 (18.8)	40 (15.7)	1.19 (0.54–2.63)	0.99 (0.041–2.51)	0.995
35+	19 (22.4)	40 (15.7)	1	1	
*Women education*					
Having no formal education	38 (44.7)	62 (24.3)	3.12 (1.58–6.19)	2.8 (1.31–6.11)^*∗∗*^	0.008
Elementary	26 (30.6)	78 (30.6)	1.42 (0.08–2.50)	1.7 (0.87–3.17)	0.127
Secondary and above	21 (24.7)	115 (45.1)	1	1	
*Number of children*					
≤4 children	38 (44.7)	146 (57.3)	1.7 (1.01–2.72)^*∗*^	1.8 (1.01–3.21)^*∗∗*^	0.043
≥5 children	47 (55.3)	109 (42.7)	1	1	
*History of abortion*					
Yes	15 (17.6)	73 (28.6)	1.9 (1.01–3.48)^*∗*^	2.3 (1.1–3.21)^*∗∗*^	0.043
No	70 (82.4)	182 (71.4)	1	1	
*Counseling service given*					
Yes	31 (36.5)	167 (65.5)	0.3 (0.18–0.51)^*∗*^	0.36 (0.21–0.64)^*∗∗*^	0.001
No	54 (63.5)	88 (34.5)	1	1	
*Decision maker to use IMP*					
Woman herself	40 (47.1)	160 (62.7)	1	1	
Other body (provider, husband)	45 (52.9)	95 (37.3)	0.53 (0.32–0.87)^*∗*^	0.65 (0.38–1.13)	0.125
*Follow-up appointment given*					
Yes	29 (34.1)	155 (60.8)	0.33 (0.20–0.56)^*∗*^	0.35 (0.21–0.62)^*∗∗*^	0.001
No	56 (65.9)	100 (39.2)	1	1	
*Side effect *					
Yes	37 (43.5)	143 (56.1)	1.7 (1.01–2.72)^*∗*^	1.25 (0.72–2.19)	0.424
No	48 (56.5)	112 (43.9)	1	1	
*Service satisfaction*					
Yes	30 (35.3)	126 (49.4)	1	1	
No	55 (64.7)	129 (50.6)	0.6 (0.34–0.93)^*∗*^	0.67 (0.35–1.12)	0.131

*Key note*.  ^*∗*^Candidate variables for multivariable analysis at *p* value ≤ 0.25. ^*∗∗*^Statistically significant at *p* ≤ 0.05 in multivariable logistic regression.

## Abbreviations

AOR: Adjusted odd ratio
COR: Crude odd ratio
CI: Confidence interval
DWSIN: Discontinuation while still in need
HEP: Health extension program
ID: Implanon discontinuation
IMP: Implanon
LARC: Long-Acting Reversible Contraceptive
MSI: Marie Stopes International
SPSS: Statistical package for social science
SSA: Sub-Saharan Africa.

## Additional Points

*Recommendation*. Based on the findings of the study the following recommendations are made. At district level: district's education office with collaboration of other concerned bodies should take into consideration addressing females education. At health facility level: the health care providers at facility level should provide appropriate preinsertion counseling according to the National Guide Line for Family Planning Service by giving emphasis on informed choice and possible expected side effects. Adequate follow-up of Implanon users by giving special attention to for younger women and those women with less than or equal to four living children should be made to increase Implanon continuation. Further research should be undertaken by mixing with qualitative study to identify more determinants of Implanon discontinuation.
